# Exophtalmie de l’œil gauche en faveur d'un carcinome adénoide kystique orbitaire: à propos d'un cas

**DOI:** 10.11604/pamj.2015.22.168.7134

**Published:** 2015-10-21

**Authors:** Adil Bouzidi, Said Iferkhass, Zine El Abidine Hansali, Mohammed Elmallaoui, Abdelkader Laktaoui

**Affiliations:** 1 Hôpital Militaire Moulay Ismail, Mekens, Maroc

**Keywords:** Cylindrome, tumeur, exophtalmie, cylindroma, tumor, exophthalmia

## Abstract

Le Cylindrome orbitaire (carcinome adénoide kystique) est une tumeur maligne d'agressivité locale et d’évolution lente. Sa localisation orbitaire aux dépens des glandes lacrymales est exceptionnelle. Nous rapportons le cas d'un patient de 51 ans consultant pour une exophtalmie gauche non axile irréductible, douloureuse; avec ptosis modéré et baisse d'acuité visuelle. Une biopsie de la tumeur par voie palpébrale supérieure a été faite, en faveur d'un un carcinome adénoïde kystique cribriforme de la glande lacrymale. Après trois mois d'un traitement conservateur chirurgical, une exentération totale a été effectuée suivie d'une radiothérapie complémentaire. Le patient a été revu tout les 3 mois en consultation durant la première année. Sur un recul de six ans, le patient ne présente pas de signe en faveur d'une récidive.

## Introduction

Le Cylindrome orbitaire (carcinome adénoide kystique) est une tumeur maligne d'agressivité locale et d’évolution lente. Il s'agit d'une tumeur épithéliale maligne développée aux dépens habituellement des glandes salivaires (sus mandibulaires et accessoires [1]. Sa localisation orbitaire aux dépens des glandes lacrymales est exceptionnelle. L'imagerie neuroradiologique est en faveur d'une tumeur au dépond de la glande lacrymale.

## Patient et observation

**Figure 1:**
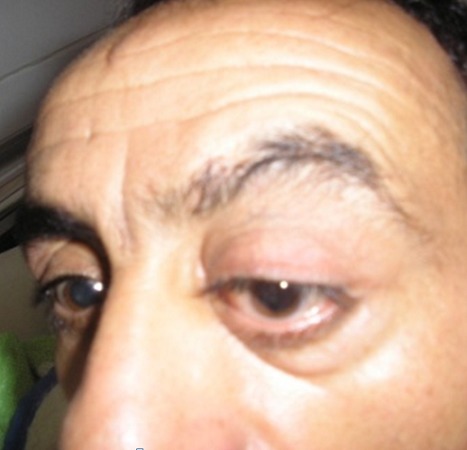
Exophtalmie de l’œil gauche avec faux ptosis

**Figure 2:**
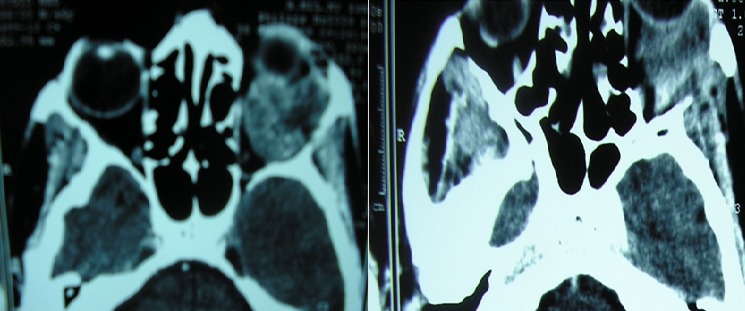
TDM orbito cérébral: un processus lésionnel intra et extraorbitaire gauche occupant les deux tiers postérieur de l'orbite et refoule le globe en bas et en avant

**Figure 3:**
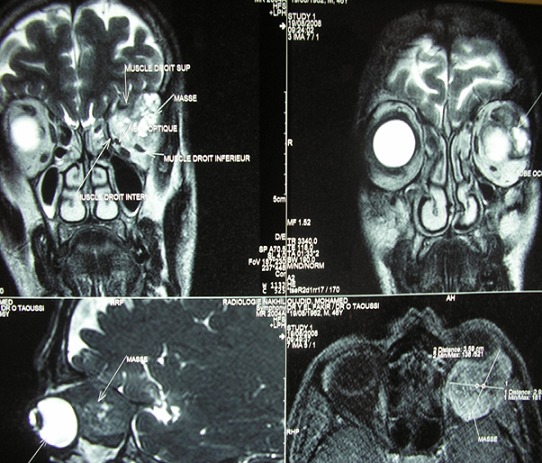
IRM orbito cérébral: une masse tissulaire hyperdense en T1 hyperdense en T2, hétérogène se rehausse fortement après injection de godalinim

**Figure 4:**
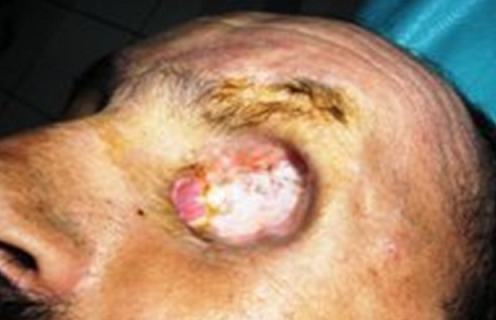
Exentération de l’œil gauche

Nous rapportons le cas d'un patient de 51 ans consultant pour une exophtalmie gauche, douloureuse; avec baisse d'acuité visuelle. A la palpation du cadre orbitaire on retrouve une masse de l'angle supéro-externe de l'orbite gauche, peu inflammatoire, peu douloureuse, de consistance dure et irréductible (Figure 1). L'acuité visuelle est de 10/10 au niveau de l’œil droit et de 4/10 au niveau de l’œil gauche. Le tonus oculaire est 13 mmHg au niveau de l’œil droit et de 12 mmHg au niveau de l’œil gauche. L'examen ophtalmologique du segment antérieur et normal. L'examen du fond d’œil montre un flou papillaire au niveau gauche avec à l'angiographie rétinienne, des drusens papillaire. Le scanner orbitaire montre un processus lésionnel intra et extraorbitaire avec une petite calcification qui se rehausse après injection du produit de contraste occupant les deux tiers postérieur de l'orbite et refoule le globe en bas et en avant (Figure 2). L'imagerie par résonance magnétique est en faveur d'une tumeur au dépond de la glande lacrymale en montrant une masse tissulaire hyperdense en T1 hyperdense en T2, hétérogène se rehausse fortement après injection de godalinim. De siège extraconique au niveau de la partie superoexterne de la cavité orbitaire. Cette masse mesure 29, 30, 35 mm de diamètre, refoule le nerf optique et le globe oculaire en avant en bas et en dehors. Elle refoule également le muscle droit inférieur en bas et le muscle droit supérieur en haut avec lyse osseuse de la paroi orbitaire externe (Figure 3). Le bilan d'extension fait d'un scanner thoraco abdomino pulvien et d'une scintigraphie osseuse est revenu normal. Une biopsie de la tumeur par voie palpébrale supérieure a été faite. L'examen anatomopathologique de la pièce opératoire révèle un carcinome adénoïde kystique cribriforme de la glande lacrymale. Une exérèse de la totalité de la tumeur a été tenté par l’équipe de neurochirurgie par voie bi coronale mais l'exérèse était incomplet vue la friabilité de la lésion, son extension et sa localisation en fer à cheval sur le nerf optique; trois mois après, une exentération totale a été effectuée suivie d'une radiothérapie complémentaire (Figure 4). Le patient a été revu tout les 3 mois en consultation durant la première année. Sur un recul de six ans, le patient ne présente pas de signe en faveur d'une récidive.

## Discussion

Le carcinome adénoïde kystique est une tumeur rare qui représente moins de 2% parmi tous les tumeurs malignes de la tète et du cou [1]. La prédominance féminine a été rapportée. Cependant, il ya des études rapportant une prédominance masculine pour ces tumeurs, et d'autres rapportent une égalité entre les deux sexes [1]. Elle est principalement une tumeur de l’âge adulte avec un pic d'incidence défini dans la quatrième à la sixième décennie de vie [1]. Le pronostic est bien meilleur chez les femmes que les hommes, ceci peut être expliqué par une grande sensibilisation du corps avec un diagnostic plus précoce et une supériorité biologique des femmes dans la réponse à un traitement [2]. Le début d’évolution est habituellement marqué par une exophtalmie ou un ptosis [3]. La douleur est fréquente: c'est un signe d'atteinte carcinomateuse surtout si elle est associée à une hypoesthésie dans le territoire du nerf frontal. La durée des symptômes avant la première consultation est généralement inférieure à 6 mois [3]. Le scanner est un examen indispensable en particulier pour le bilan d'extension [2, 4]. Il montre la tumeur au niveau de la loge lacrymale, ses mensurations, les marges de la lésions qui peuvent paraître irrégulières, l'existence d'une érosion osseuse qui peut être précoce, et la présence ou non de calcifications. L'IRM a pour intérêt de détecter précocement les tumeurs de petite taille et d'objectiver l’étendue de la propagation tumorale, mais elle reste, comme pour le scanner, incapable de différencier entre une tumeur maligne et bénigne [2, 5]. Seul l'examen anatomopathologique permet un diagnostic de certitude. Trois grandes formes anatomopathologiques ont été identifiées: tubulaire, cribriforme et solide [5, 6]. Plusieurs facteurs pronostiques ont été suggérés pour ACC de la tête et du cou. Sous-type histologique et le grade histologique sont parmi eux. Plusieurs études rapportent un pronostic favorable pour la forme du CAC tubulaire et criblée, que pour le type solide. L'Invasion périneurale a été aussi, identifiée comme un facteur pronostique défavorable.

Le traitement est guide par la classification TNM [5]. Il est essentiellement chirurgical: biopsie exérèse par orbitotomie latérale si pas d'atteinte métastatique [5, 6]. La radiothérapie externe et la chimiothérapie peuvent être utiles dans certaines circonstances particulières. Le taux de récidives reste élevé [1, 3]. Il est douteux que la radiothérapie adjuvante a de la valeur dans la prévention de la récidive locale. Malgré le fait que 83,3% des patients avec des marges lésionnelles positives, avaient développé une récidive locale, la radiothérapie a probablement contribué à la réalisation de contrôle local dans le 16,7% restants des tumeurs avec des marges positives [1]. En général, la radiothérapie comme la seule modalité de traitement semble être insuffisante pour ACC. Cependant elle reste une modalité adjuvante à la chirurgie pour améliorer le contrôle local de la tumeur, surtout quand il y a une tumeur résiduelle microscopique [1]. La décision d'effectuer une exentération orbitaire est généralement motivée par l'atteinte de l'apex orbital objectivé radiologiquement, ou dans certains cas, par l'extension au-delà de l'orbite dans les sinus et le parenchyme cérébral [1, 5, 6]. La décision, chez notre patient, de réaliser une exentération suivie d'une radiothérapie est prise devant le récidive malgré une chirurgie conservatrice et devant l'attente de l'apex et l'os. La détection de métastases aux poumons ou à d'autres organes chez les patients asymptomatiques ne sont pas une indication pour un traitement ultérieur, comme la radiothérapie et la chimiothérapie puisque elles sont inefficaces pour la gestion des dépôts du CAC secondaires. Mais elles ont un certain avantage palliatif en cas de maladie métastatique symptomatique [1]. Le risque de récidive locale était plus élevé chez les patients traités par chirurgie conservatrice, par opposition à exentération orbitaire et il était plus élevé chez les patients qui ne reçoivent pas la radiothérapie postopératoire [5]. Après un recul de six ans, notre patient ne présente aucune récidive locale ni générale et nous suggérons que l'exentération suivie de radiothérapie reste le traitement de référence devant le grade T3 avec atteintes des autres structures orbitaires et la paroi osseuse.

## Conclusion

Le cylindrome orbitaire est une tumeur rare avec une croissance lente et une survie de 69% à 5 ans. Les récidives locales sont fréquentes. La localisation orbitaire est exceptionnelle et un diagnostic précoce est nécessaire. Comme dans l'ensemble des lésions orbitaires, lorsque la clinique ou l'imagerie n'est pas typique ou évocatrice d'une lésion, une biopsie orbitaire doit être envisagée afin d’établir le diagnostic anatomopathologique.
